# Metastatic L1 root compression presenting as an inguinal pain: A case report

**DOI:** 10.1002/ccr3.4286

**Published:** 2021-05-19

**Authors:** Babak Mirzashahi, Farzad Vosoughi, Saied Besharaty, Sadegh Hasani Satehi

**Affiliations:** ^1^ Orthopedic Surgery Department Imam Khomeini Hospital Tehran University of Medical Sciences Tehran Iran

**Keywords:** case report, laryngeal cancer, radiculopathy, spinal metastasis

## Abstract

As the spine is the third most common place for metastatic involvement, spinal examination is essential in patients with a known history of cancer, even in those with no related complains (ie. Backpain).

## Graphical abstract

A missed spinal metastasis may lead to devastating complications (e.g. spinal cord compression).

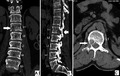

## 1 INTRODUCTION

We report a spinal metastatic patient with an atypical presentation of inguinal pain. He was managed with L1 laminectomy and posterior instrumentation. After the surgery, his pain was significantly relieved. This study underscores the importance of spinal examination in those with a known tumor.

The spine is the most common destination for tumor metastasis after lung and liver,^1^ and spine metastases are the most frequent spinal tumors.^2^ Up to 40 percent of patients with malignant tumor are shown to have spine metastasis.^3^ Recognition of spinal involvement in those with cancer is important as the spine involvement is the first presenting finding in around 10 percent of patients with cancers.^2^ Also, symptomatic spinal cord compression due to spine metastasis can lead to an irreversible spinal cord injury after only a week in around one‐third of cases.^4^ Recently, due to improvement in the diagnostic and therapeutic armamentarium, the prevalence of spinal metastasis has increased significantly.^3^, ^5^, ^6^ So, the need to appropriately diagnose and manage spinal metastasis is strongly felt.

In the following, we present a case of a metastatic vertebral (L1) involvement referred to our clinic with an atypical presentation of severe inguinal pain. Then, we further delineate the diverse clinical manifestation of metastatic spinal diseases reported in the literature.

## 2 CASE REPORT

**FIGURE 1:**
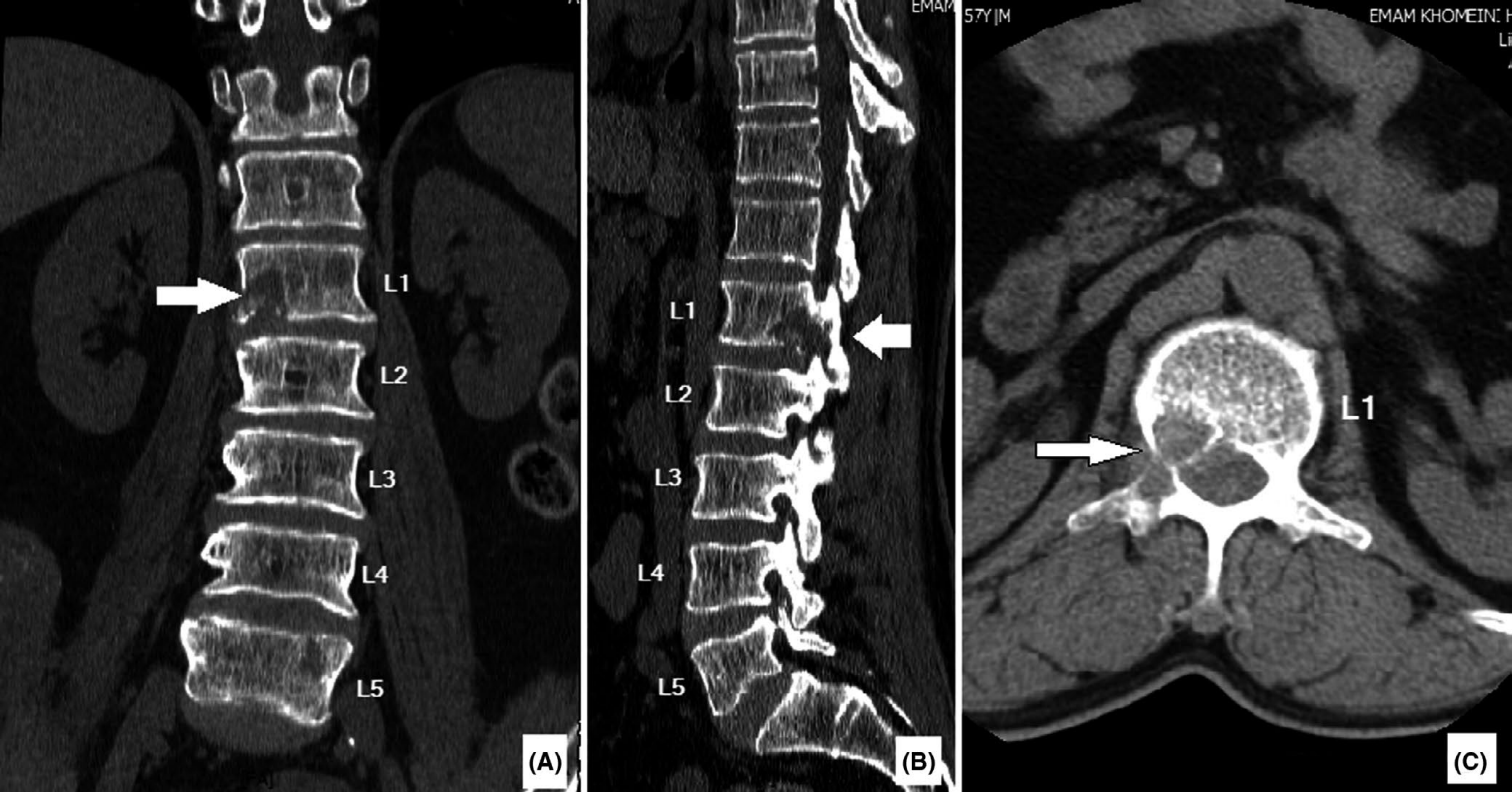
Preoperative CT scan of our patient. Coronal A, sagittal B, and axial C reconstructions illustrate a lytic lesion in the body and right‐sided pedicle of the first lumbar vertebra

**FIGURE 2:**
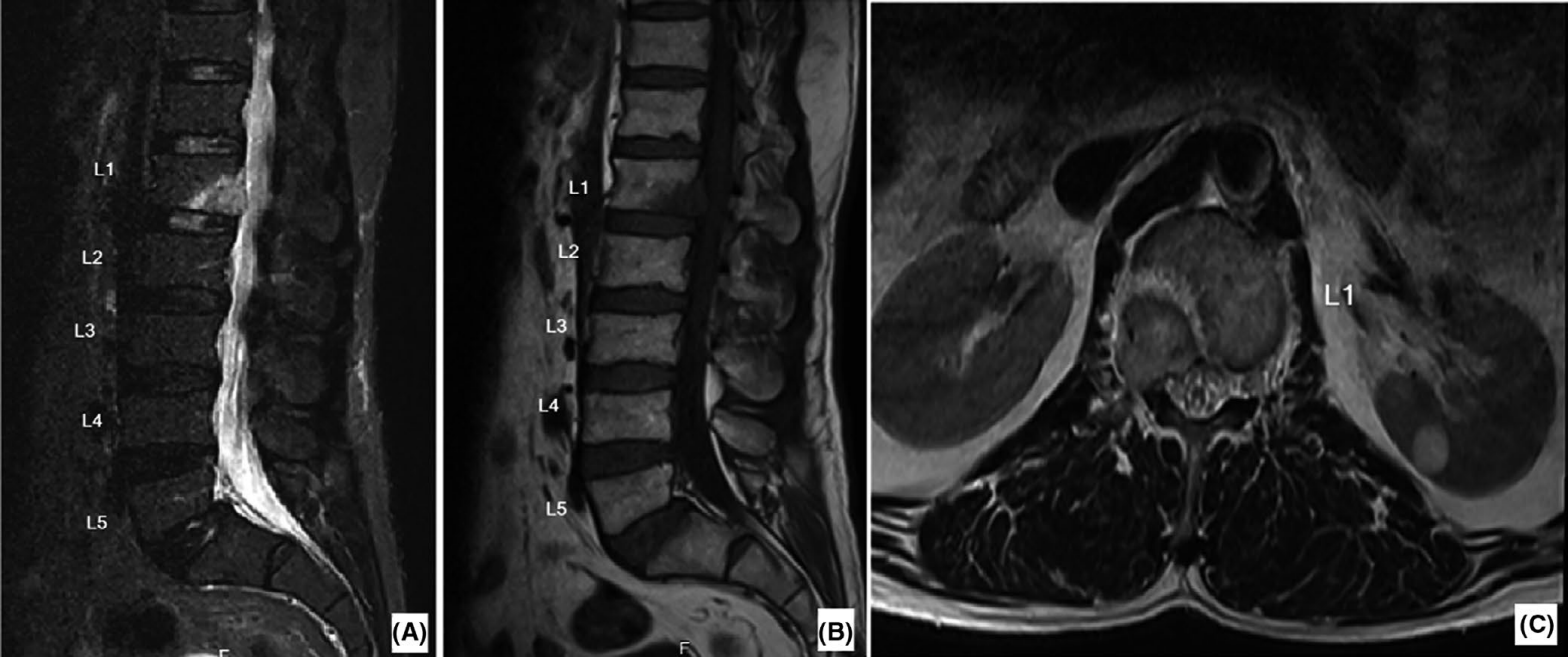
Magnetic resonance imaging of the presented patient. It illustrates a spinal lesion which is high signal in T2 A, and low signal in T1 B. The axial reconstruction demonstrates an extradural mass compressing the first lumbar nerve root C

A 64‐year‐old man, who had undergone laryngectomy to treat laryngeal cancer 12 years ago, was referred to our clinic with severe pain at right inguinal region preventing him from walking. He did not report any radiation of his pain to other areas. The pain was progressive and started from 1 year ago. It was exacerbated by walking. He mentioned that his pain was not alleviated with rest and does not allow him to sleep. He had been evaluated by different specialists. He had taken many analgesics; however, none of them had relieved his chief complaint. His family and social history were insignificant. During physical examination, he walked with difficulty. In supine position, he tended to keep his right hip in 30 degrees of hip flexion. His right hip extension in 0‐30 flexion was painful. His right knee range of motion was normal. No sign of scar, mass, erythema, or tenderness was detected in his right inguinal area. No tenderness was detected palpating his right sacroiliac joints, posterior superior iliac spine, iliac crest, anterior superior iliac spine, or symphysis pubis. Meanwhile, during spine examination, tenderness on the patient's spine at thoracolumbar junction was noted. His previous workup included pelvic X‐ray (AP), right hip X‐ray, and MRI, all of which were insignificant. Due to his spinal tenderness, a lumbosacral plain radiography, lumbosacral computed tomography (CT) [Figure 1], and magnetic resonance imaging (MRI) [Figure 2] scan were performed. On spine plain X‐ray, the right pedicle at the level of L1 was not visible (winking owl sign). CT scan revealed a lytic lesion in the posterior half of the L1 vertebral body and the L1 right pedicle. The lesion involved the inferior body cortex. On MRI, the lesion turned out to be a mass in the posterior L1 body and its right pedicle obliterating the right intervertebral foramen. It was hyposignal on T1 and hypersignal on T2. On bone scan, foci of hyper absorption on several of his ribs and lumbar vertebrae were present.

**FIGURE 3:**
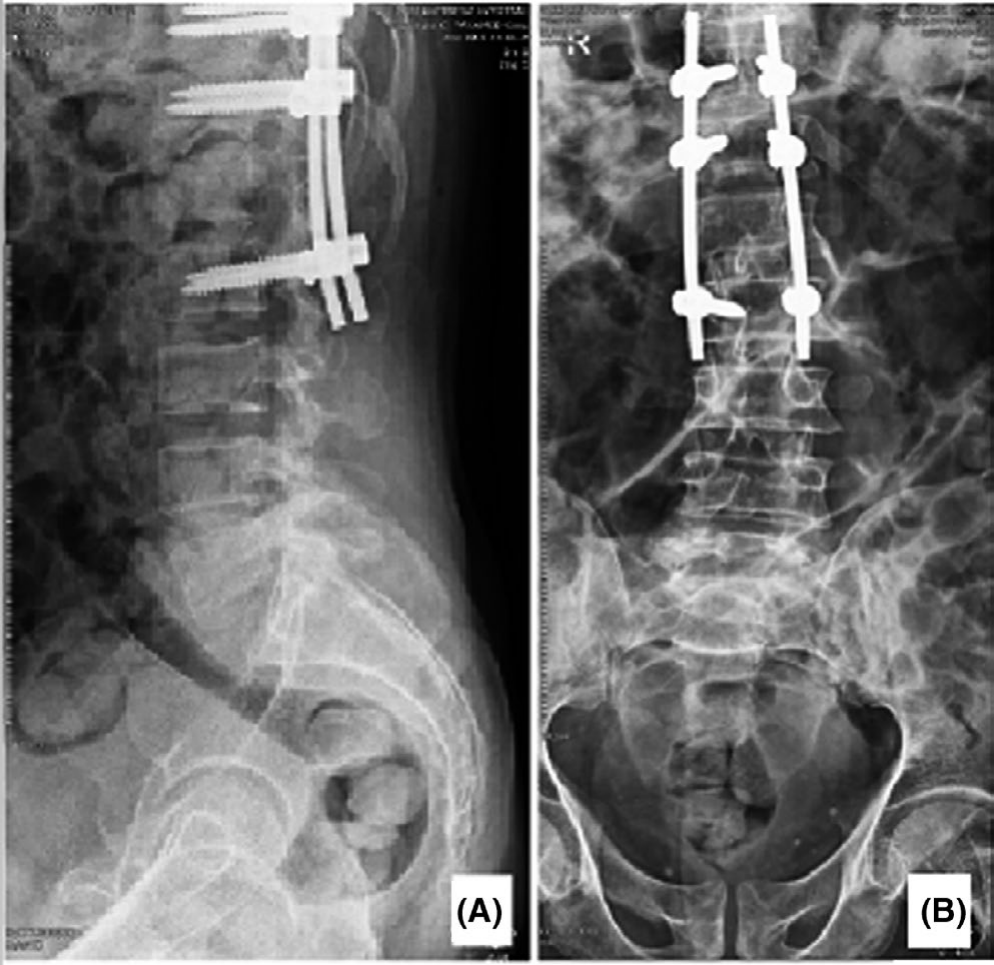
Postoperative plain lumbosacral X‐ray of the presented patient. Lateral A, and anteroposterior B, views are demonstrated

As a result, our patient was managed with L1 vertebral laminectomy and root decompression. The mass obliterated the right L1 intervertebral foramen. Thus, we had to excise his right L1 pedicle and right L1 superior facet in order to visualize the right L1 intervertebral foramen and decompress the right L1 root through its course. The spinal lesion was biopsied and sent for pathology. As the L1 vertebrae were unstable after excising its right pedicle and superior facet, posterior instrumentation was performed to enhance the spinal stability [Figure 3]. Interestingly, the day after surgery inguinal pain was relieved significantly, and the patient succeeded to go out of bed with a thoracolumbosacral orthosis (TLSO). The pathology reported metastatic adenocarcinoma showing papillary configuration with pulmonary origin. Our patient was referred to a radiotherapist and oncologist for further radiotherapy/chemotherapy treatment. After referring him to the oncologist, it was revealed that he had also foci of lung metastases and managed with chemotherapy. He visited our clinic regularly for follow‐up at 2 weeks, 6 weeks, 12 weeks, 24 weeks, and 1 year postoperatively. After 1 year postoperatively, our patient ambulated independently, and his inguinal pain was relieved and had no operative complication.

## 3 DISCUSSION

The most common complaint in spinal metastatic cases is pain followed by weakness, sensory impairment, and bladder or bowel dysfunction.^2^ Spinal metastatic cases may have local, mechanical referral, or neuropathic pain. Local pain has a deep aching nature, exacerbates while the patient sleeps, and improves by nonsteroidal anti‐inflammatory drugs. Mechanical pain is due to the spinal instability. It enhances with changing of position and decreases with lying down. Neuropathic pain is seen in intradural metastasis and has a burning quality. Finally, referral pain is a result of neural root compression caused by the tumor and has a sharp or stabbing nature.^6^

Sciubba et al^6^ stated that only one type of pain may present in spinal metastasis. This is in accordance with our finding, as our patient had a referral pain to the inguinal L1 dermatome without any history suggestive of neuropathic or mechanical pain.

As the spine is the third most common place for metastatic involvement, it is recommended to maintain a high index of suspicion for this diagnosis when managing patients with a known tumor. A back or neck pain in those with a known tumor should be considered spinal metastasis until proven otherwise.^2^

Our patient had no complaint of cervical or back pain; however, on examination, a thoracolumbar tenderness hinted the medical team to evaluate the spinal column. Therefore, this case scenario underscores the importance of spinal examination in those with a known tumor, in order not to miss the spinal metastasis in these patients.

Shaohui He et al^5^ reported the local pain and night aggravating pain as the symptom with most negative and positive predictive value for diagnosing spinal metastasis, respectively. Our patient did not complain of a local spinal pain and, however, had a local spinal tenderness in the thoracolumbar area.

Symptomatic spinal metastasis most commonly is seen in thoracic spine followed by cervical and lumbar vertebrae.^2^, ^3^ In a study performed among patients with breast cancers, the least common spinal metastasis revealed to be thoracolumbar, lumbosacral, and sacral vertebrae.^7^ In our case, the thoracolumbar area (L1) was involved.

Many studies have demonstrated that symptoms of spinal cord compression caused by spinal metastasis can be effectively improved by spinal decompression surgery. Our finding was similar, as our patient's pain significantly declined immediately after the surgery. Thus, operative management not only may provide spinal metastatic cases with a chance of tumor growth control but also has even more important role in improving the quality of life in these cases.

As a missed spinal metastasis may lead to spinal cord compression, early diagnosis is of paramount importance. This study demonstrates that a spinal metastatic patient needing urgent management may not complain of a neck/back pain. Given the relative high possibility of spinal metastatic involvement, routine examination of vertebral column in patients with malignancy may help the physician in order not to miss spinal involvement. However, future studies on large groups of these patients may give a better understanding of the treatment of spinal metastatic patients.

## 4 CONCLUSION

This study highlights the importance of correct history taking and physical examination in managing patients with a known tumor. Complaint of back pain or finding of spinal tenderness should alarm the physician to consider the possibility of spinal metastasis in any patient with a previous history of cancer. Also, hip pain in any patient should alert the physician to a possibility of upper lumbar root compression.

## CONFLICT OF INTEREST

The authors have completed the ICMJE uniform disclosure form. The authors have no conflicts of interest to declare.

## AUTHOR CONTRIBUTIONS

BM: conducted the surgery and supervised preparing the manuscript. FV: reviewed literature and wrote the manuscript. SB: edited the manuscript and collected the patient's data. SHS: edited the manuscript.

## ETHICAL APPROVAL

The authors are accountable for all aspects of the work in ensuring that questions related to the accuracy or integrity of any part of the work are appropriately investigated and resolved. All procedures performed in studies involving human participants were in accordance of ethical standards of the institutional and/or national research committee(s) and with the Helsinki Declaration (as revised in 2013). Written informed consent was obtained from the patient for publication of the case report and any accompanying images.
